# Betalain, Acid Ascorbic, Phenolic Contents and Antioxidant Properties of Purple, Red, Yellow and White Cactus Pears

**DOI:** 10.3390/ijms12106452

**Published:** 2011-09-28

**Authors:** María Teresa Sumaya-Martínez, Sandra Cruz-Jaime, Eduardo Madrigal-Santillán, Juan Diego García-Paredes, Raquel Cariño-Cortés, Nelly Cruz-Cansino, Carmen Valadez-Vega, Leonardo Martinez-Cardenas, Ernesto Alanís-García

**Affiliations:** 1 Secretary of Research and Graduate Studies, Autonomous University of Nayarit, Ciudad de la Cultura “Amado Nervo”, Boulevard Tepic-Xalisco S/N. Tepic, Nayarit, Mexico; E-Mails: digapa1@hotmail.com (J.D.G.-P.); leonarm2@yahoo.com.mx (L.M.-C.); 2 Institute of Health Sciences, Autonomous University of Hidalgo State, Ex-Hacienda de la Concepción, Tilcuautla, 42080 Pachuca de Soto, Hgo, Mexico; E-Mails: crzjms@msn.com (S.C.-J.); eomsmx@yahoo.com.mx (E.M.-S.); raque_nat@hotmail.com (R.C.-C.);cruz_cansino@hotmail.com (N.C.-C.); m.valadezvega@lycos.com (C.V.-V.); ernesto_alanisgarcia@hotmail.com(E.A.-G.)

**Keywords:** antioxidant activity, cactus pears, betalain, phenolic compounds, ascorbic acid

## Abstract

Commercialization of cactus pears based on their antioxidant properties can generate competitive advantages, and these can turn into business opportunities and the development of new products and a high-value ingredient for the food industry. This work evaluated the antioxidant activities (1,1-diphenyl-2-picrylhydrazyl radical-scavenging, protection against oxidation of a β-carotene-linoleic acid emulsion, and iron (II) chelation), the content of total phenolic compounds, ascorbic acid, betacyanin, betaxanthin and the stability of betacyanin pigments in presence of Cu (II)-dependent hydroxyl radicals (OH•), in 18 cultivars of purple, red, yellow and white cactus pear from six Mexican states. Our results indicated that the antiradical activities from yellow and white cactus pear cultivars were not significantly different (*p* < 0.05) and were lower than the average antiradical activities in red and purple cultivars. The red cactus pear from the state of Zacatecas showed the highest antioxidant activity. The free radical scavenging activity for red cactus pears was significantly correlated (*p* < 0.05) to the concentration of total phenolic compounds (*R*^2^ = 0.90) and ascorbic acid (*R*^2^ = 0.86). All 18 cultivars of cactus pears studied showed significant chelating activity of ferrous ions. The red and purple cactus pears showed a great stability when exposed to OH•.

## 1. Introduction

Plants from the genus *Opuntia* are the most abundant of the Cactaceae family, grown throughout the Americas as well as the central area of the Mediterranean, Europe, Asia, Africa, and Australia. *Opuntia* species display flattened stems called “pencas” or cladodes. The cactus pear is the fruit of this plant (*Opuntia* spp.). The fruit is an oval berry with a large number of seeds and a semi-hard rind with thorns, which may be grouped by fruit colors: red, purple, orange-yellow and white. The fruits with white pulp and green rind are preferred for consumption as food, and their domestic production corresponds to almost 95% of the total production. Mexico is the main producer of cactus pears and accounts for more than 45% of the worldwide production; however, only 1.5% of this production is exported [1–3].

A viable strategy to increase the competitiveness of the Mexican cactus pear in national and international markets is the innovation and creation of new high value-added products. This could be achieved by determining the nutritional and functional properties that differentiate the Mexican cactus pear from analogous products. In addition, providing functional products for a market in constant growth would offer a key competitive advantage and would allow the producers to diversify its commercialization, not as fresh fruit only, but also as an ingredient or high-value additive for the food industry. A commercialization of the cactus-pear based on its antioxidant properties could generate competitive advantages that may turn into business opportunities and the development of new products [4].

Recent studies in the varieties of European and Asian cactus pears have shown notable antioxidant activities that significantly reduce oxidative stress in patients and may help in preventing chronic pathologies. Betalain pigments contained in these cactus pears have shown beneficial effects on the redox-regulated pathways involved in cell growth and inflammation [5–8]. Several studies have suggested that the total antioxidant activity of the cactus pear is due to its content of vitamin C, polyphenolics, flavonoid compounds (e.g., kaempferol, quercetin, and isorhamnetin), pigments (betalains) and taurine [9–16]. Betalains are water-soluble pigments. Two betalain derivatives are present in cactus-pears: betacyanin, which gives the red-purple color, and betaxanthin, which gives a yellow-orange color. These pigments show important antioxidant activities without toxic effects in humans [16–18].

In addition, a neuroprotector activity against oxidative damage induced in cultures of rat cortical cells has been attributed to the cactus pear flavonoids [19]. Another beneficial effect of the fruit was observed in the prevention of stomach ulcers through the stimulation of prostaglandin production: cactus pear promoted mucous secretion of bicarbonate, involved in the protection of gastric mucosa [10].

On the other hand, their contents of natural antioxidants has raised interest in the use of cactus pears as substitute for synthetic antioxidants, such as butylhydroxytoluene (BHT), butylhydroxyanisole (BHA) and butylhydroxyquinone (BHQT), added to foods susceptible to lipidic oxidation. Nevertheless, some studies have demonstrated that these synthetic antioxidants have cytotoxic effects [20]. The use of cactus pear extracts as a source of antioxidants could be a promising alternative for the food industry. Then again, this fruit may be a good source of color, phenolic compounds, fiber, vitamins, and polyunsaturated fatty acids, all of which add to its high value [21,22].

The objective of this study was to evaluate the antioxidant activities (1,1-diphenyl-2-picrylhydrazyl radical-scavenging, protection against oxidation of a β-carotene-linoleic acid emulsion, and iron(II) chelation), the content of total phenolic compounds, ascorbic acid, betacyanin, betaxanthins and the stability of betacyanin pigments in presence of Cu(II)-dependent hydroxyl radicals (OH•), in 18 cultivars of cactus pear from six Mexican states.

## 2. Experimental Section

### 2.1. Chemicals

All chemicals and solvents used in this study were of reagent grade and purchased from Sigma-Aldrich, St. Louis, MO, USA and J.T. Baker, USA. Freshly prepared redistilled water was used in the present study.

### 2.2. Plant Materials

Samples corresponded to the 18 cultivars of purple, red, yellow and white cactus pear predominantly produced in Mexico’s central plateau. The botanical families for the red and white cactus pears were *O. ficus-indica* (L.) Mill and *O. albicarpa*, respectively; for the Picochulo, Huesona and Milpa Alta yellow cactus pears were *O. megacantha*, *O. albicarpa* and *O. ficus-indica* respectively; for Torreoja purple cactus pear were *O. megacantha*, for the rest of purple cactus pears the botanical family is unknown.

Fruits were harvested in six Mexican states: Hidalgo, Puebla, Guanajuato, Jalisco, Zacatecas and the State of Mexico. The fruits were donated by the main region producers and were selected in their commercial maturity stage (Table 1). The juice was extracted from the pulp of nine fruits of each cultivar, centrifuged at 5000 rpm for 10 min at 4 °C, filtered through a 0.45 μm millipore hydrophilic PVDF syringe filter (25-mm diameter) and frozen at −70 °C. For each juice, degrees Brix (°Bx), pH, the concentration of total phenolic compounds, ascorbic acid, betalain contain as well as its antioxidant properties, were determined by triplicate.

### 2.3. Determination of Betalain Content

Betacyanins and betaxanthins content was determined according to Castellar *et al.* [16] and Stintzing *et al.* [23], were reported like mg equivalent betanin/L and mg equivalent indicaxanthin/L, respectively. Betacyanins were detected at 535 nm and betaxanthins at 484 nm, according to the next equation:

Betacyanins or betaxanthins content [mg/L]=[(A×DF×MW×1000/∈×1)]

where: A = absorbance at 535 or 480 nm, DF = dilution factor, MW = molecular weight, ∈ = extinction coefficient, l = width of the spectrophotometer cell (1 cm)

For betacyanin the extinction coefficient is 60,000 L/(mol cm) and MW = 550 g/mol. For betaxanthins the extinction coefficient is 48,000 L/(mol cm) and MW = 308 g/mol.

### 2.4. Determination of Total Phenolic Content

Total phenolic content of the juice was determined according to Georgé *et al.* [24]. Briefly, 0.10 mL of the juice (a different dilution factor in water was utilized from each sample) was added into 0.500 mL of 1:10 diluted Folin–Ciocalteu reagent and 0.400 mL of sodium carbonate solution (at 7.5%) was added. The absorbance of the mixture was measured at 765 nm in a microplate reader (PowerWave XS, Biotek instruments, USA), after incubation for 0.5 h at room temperature. Gallic acid was used as a reference standard and the results were expressed as milligram gallic acid equivalent (mg GAE)/L of juice.

### 2.5. Determination of Acid Ascorbic Content

Acid ascorbic content of the juice was determined according Dürüst *et al.* [25]. Briefly, 0.10 mL of the juice (a different dilution factor in acid oxalic at 0.4% was utilized from each sample) was added into 0.100 mL of acetate buffer and 0.800 mL of DCPI (2,6-dichloroindophenol sodium) was added. The absorbance of the mixture was measured immediately at 520 nm in a microplate reader. Acid ascorbic was used as a reference standard and the results were expressed as mg acid ascorbic/L of juice.

### 2.6. Free Radical Scavenging Activity

Antiradical activity was measured with DPPH• (1,1-diphenyl-2-picrylhydrazyl radical) as described by Morales and Jimenez-Perez [26]. A 7.4 mg/100 mL ethanolic solution of the stable DPPH• radical was prepared. Then 100 μL of the samples were taken into vials, and 500 μL of DPPH• solution were added. The solution was stirred in a vortex and centrifuged at 10,000 rpm for 5 min at 4 °C after standing one hour at room temperature. Finally, absorbance was measured at 520 nm in a microplate reader, and μmol equivalents of Trolox/L (μmol ET/L) were obtained.

### 2.7. Antioxidant Activity

Antioxidant activity based on the protection against oxidation of a β-carotene-linoleic acid emulsion under the combined effect of light, oxygen and heat was determined according to Vankar *et al.* [27]. The emulsion was prepared by placing 2 mL of β-carotene solution (0.2 mg/mL in chloroform) in a flask, evaporating the chloroform at 40 °C for 15 s using a rotary evaporator, and then adding 40 mL of linoleic acid, 400 mL of Tween-20, and 100 mL of deionized water. For the emulsion to be formed, the ball flask was shaken vigorously in a rotary evaporator for one min. 200 μL-samples were placed in test tubes by triplicate, and 5 mL of the emulsion was added to each tube, stirred in a vortex, and then immersed in a thermostatic water bath at 50 °C. BHT solution (0.1%) in deionized water was used as a control standard for protection against oxidation. Readings were recorded at 0, 15, 30, 60, and 90 min. Absorbance was measured at 470 nm in a microplate reader. The antioxidant activity of the samples was calculated using the following equation:

% Protection=(AE90/AE0)×100

where AE90 = Absorbance of the emulsion at time 90 min, and AE0 = Absorbance of the emulsion at time 0.

### 2.8. Chelating Activity of Ferrous Ions

Chelating activity was determined as described by Gulcin *et al.* [28] with slight modifications. 100 μL-samples were placed in vials, and 50 mL of ferric (II) chloride solution (2 mM) and 450 mL of methanol were added. The mixture was mixed by vortexing and then left for 5 min at room temperature. Then 200 mL of ferrozine (5 mM) was added, and the mixture was vortexed again and allowed to settle for 10 min at room temperature. Absorbance was read at 562 nm in a microplate reader. Deionized water was used under the same conditions as a control. The chelating activity was calculated using the following equation:

Chelation%=((AMC-AM)/AMC)×100

where AMC = Absorbance of the control sample and AM = Absorbance of sample.

### 2.9. Stability of Betacyanin Pigments in the Presence of Hydroxyl Radicals (OH•)

This technique was applied only to red and purple varieties, because it determined the speed of discoloration or bleaching of betacyanins in the presence of OH• radical. Based on the Fenton reaction, H_2_O_2_ is decomposed into OH• (the most reactive oxygen radical) and OH^−^ in the presence of transition metals (e.g., Fe and Cu). Two-hundred-microliters of tuna extract, 88 μL of H_2_O_2_ (3%) and 1770 μL of 20 mM phosphate buffer at pH 6.8 were placed into 3-mL test tubes. Tubes were incubated at 30 °C in a temperature-controlled bath for 10 minutes in complete darkness. Then, 10 μL of CuSO_4_ were added at different concentrations (from 50 to 6000 μM), and the absorbance was measured using a spectrophotometer at 535 nm every 7 seconds in a 30-min period. A kinetic curve for the bleaching of betacyanin in the presence of OH• radical was obtained.

Bleaching kinetics were adjusted to the exponential function, and function *y* = Ae^−b^*^x^* was fitted to the curve to calculate the speed of bleaching (b), based on the concentration of CuSO_4_.

## 3. Results and Discussion

Samples correspond to the 18 cultivars of purple, red, yellow and white cactus pear produced in Mexico’s central zone (Table 1). The observed variation in pH value for each cultivar of cactus pear may be due to differences in the maturity of the fruit from each batch. The most common pH was 6.17 according to Felker *et al*. [29], the pH and acidity for ripen cactus pears is between 5.6 and 6.5.

Obtained pH values were similar to those reported by Stintzing *et al.* [23] for nine cactus varieties. For *O. ficus-indica* [L.] Mill and *O. robusta* Wendl, pH values herein reported were in the 5.6–6.5 range. In another study by Gurrieri *et al.* [30] analyzing white, red and yellow *O. ficus-indica* fruits, a mean pH value of 6.4 was obtained. Both studies considered pH as a good indicator for the fruit maturity. Therefore, these values are less acidic than prickly pear juices reported by Chavez-Santoscoy *et al.* [22] and Díaz-Medina *et al.* [31], where the pH’s ranging from 4.27 to 5.46 and 3.32, respectively.

Average °Bx value of the 18 varieties of prickle pear was 13.7 ± 0.6%. The highest °Bx value was found in red cactus pears from Zacatecas, 15.1 ± 0.1%. No significant differences (*p* < 0.05) were observed between the extracts from purple, red, yellow and white cultivars of cactus pears.

According to the Mexican standard PC-046-2005, °Bx value of a commercial-quality cactus pear should not be less than 12%. Based on the °Bx values of the extracts from all the analyzed cultivars of cactus pears, all extracts showed commercial maturity. In a study by Stintzing *et al.* [23] with several pear varieties, the average °Bx values from higher to lower were reported in red, white, purple and orange cactus pears (14.8, 14.2, 12.8, and 12.6%, respectively), values being similar to those shown in this study. Changes in nutrient concentration, °Bx and pH may be due to the physical and chemical traits of the cactus-pears localities of origin, including the variety, species, state of maturation, crop conditions, climate and the composition of the soil where they were cultivated [1,10,32].

### 3.1. Betalain Content

**Figure 1:**
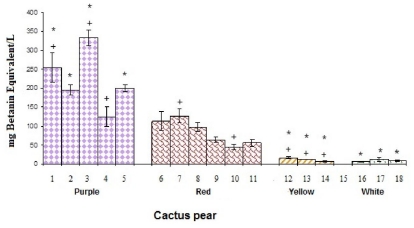
Betacyanin content of cactus pear studied cultivars. * denotes statistical significance at *p* < 0.05 among all studied *cultivars* of cactus pears. + indicates statistical significance at *p* < 0.05 among the cultivars of cactus pears in the same color.

Figure 1 shows the betacyanin concentration (BCC) for the studied cactus pears cultivars. There was no significant difference (*p* < 0.05) in the BCC between yellow and white fruits, and mean value was far below those obtained for red and purples fruits. In the red cactus pear group, the varieties with greater BCC were red Vigor from Puebla and Hidalgo (with an average 120 ± 20 mg betanin equivalents/L). In the purple group, greater concentrations of BCC were obtained in the Púrpura and Profundo cultivars from Hidalgo (254.28 ± 39.2 and 333 ± 21 mg betanin equivalents/L, respectively).

In addition, it was observed that between purple cultivars San Martín from Hidalgo and Puebla (same cultivar but from different state), BCC was significantly different. Therefore, BCC was also influenced by the crop conditions and the rate of maturation at the time of harvesting [29,32]. Chavez-Santoscoy *et al.* [15] and Stintzing *et al.* [23] reported mean values of BCC for the white, orange and red cultivars similar to the values for *O. ficus-indica* (L.) Mill and *O. robusta* Wendl obtained in this work; nevertheless, for the purple cultivar the value observed by Stintzing *et al.* [23] was higher (431 ± 1 mg of betanin equivalents/L) than the value obtained in this study for the purple cactus pear Profundo from Hidalgo (324.31 ± 19 mg of betanin equivalents/L). The analyzed cactus pears juices showed similar tendency with the reported values: the purple cactus pears have greater BCC.

**Figure 2:**
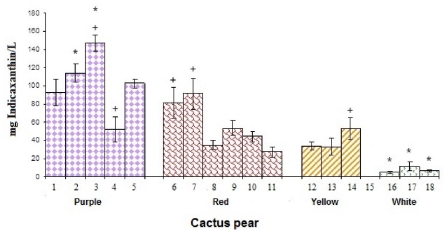
Betaxanthin content of cactus pear studied cultivars. * denotes statistical significance at *p* < 0.05 among all studied cultivars of cactus pears. + indicates statistical significance at *p* < 0.05 among the cultivars of cactus pears in the same color.

The betaxanthin concentrations (BXC) ranged for 8 to 147 mg indicaxanthin/L, the purple cactus pears had the greater BXC concentrations. The highest content corresponded to purple cactus pear Profundo from Hidalgo (Figure 2). It was observed that between purple cultivars San Martín from Hidalgo and Puebla (same cultivar but from different state) BXC was significantly different (*p* < 0.05), These BXC content were in the same order that those published by Stintzing *et al.* [23]: purple cactus pear > red cactus pear > yellow cactus pear > white cactus pear.

### 3.2. Concentration of Ascorbic Acid (CAA)

**Figure 3:**
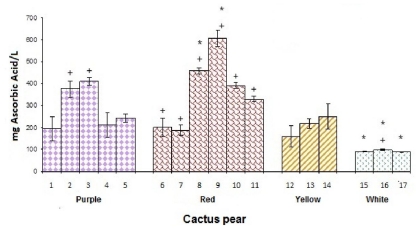
Ascorbic acid content of cactus pear studied cultivars. * denotes statistical significance at *p* < 0.05 among all studied cultivars of cactus pears. + indicates statistical significance at *p* < 0.05 among the cultivars of cactus pears in the same color.

The cultivars of red cactus pears from Zacatecas (Lirio and Lisa) had the highest levels of CAA among the tested cactus pear cultivars, with an average of 533 ± 105 mg/L (Figure 3). With regard to the purple cactus pears, Profundo from Hidalgo and Torreoja from Jalisco showed an average CAA of 395 ± 25 mg/L, significantly higher (*p* < 0.05) than the other cultivars of purple pears analyzed. It should be noted that the cultivars of white pears had the lowest mean CAA levels in the study (92 ± 2 mg/L). There were no significant differences (*p* < 0.05) between yellow cultivars of cactus pear (average 209 ± 43 mg/L), red cultivar Vigor from Puebla and Hidalgo (average 194 ± 9 mg/L) and purple cultivar San Martín from Hidalgo and Puebla (average 215 ± 25 mg/L), this indicates that CAA in the present study does not correlate with the color of a group of cactus pears.

Moreover, cactus pears of the same cultivar but from a different state such as red cultivar Vigor from Puebla and Hidalgo showed no significant difference (*p* < 0.05) in CAA. However, red cultivar Lisa from Zacatecas had 35% more CAA than that from Guanajuato. In addition, purple cultivar Profundo from Hidalgo almost doubled the concentration of CAA with respect to the purple cultivar Púrpura from the same state. Galati *et al.* [10] reported that the average CAA in the juice from yellow cactus pears was 269 ± 48 mg/L, which is slightly higher than the values obtained in this work (209 ± 43 mg/L). In another study that evaluated different-colored cultivars *O. ficus-indica* (L.) Mill and *O. robusta* Wendl, the values of ascorbic acid, determined by HPLC, were 51.1, 70.2, 67.9 and 95.4 mg/L for the white, orange, red and purple cultivars, respectively [23]. Given the different determination methods, the values obtained in some previous studies are not comparable with those reported in this paper, although trends were similar. However, the highest averages in this study were observed in juice from red cultivars. Such differences, as well, could be linked to crop conditions, as the *Opuntia* growing up under limited conditions of soil and water could change its composition, especially according to the fruit cultivar [29,32,33].

### 3.3. Concentration of Total Phenolic Compounds (TPC)

**Figure 4:**
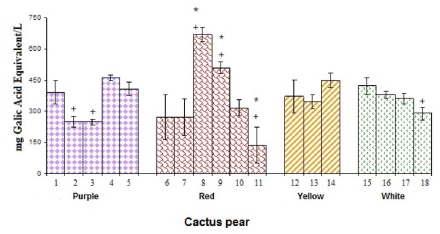
Total phenolic contents of cactus pear cultivars. * denotes statistical significance at *p* < 0.05 among all studied cultivars of cactus pears. + indicates statistical significance at *p* < 0.05 among the cultivars of cactus pears in the same color.

Red cactus pear Lirio and Lisa from Zacatecas showed the highest TPC value (670 ± 33 and 509 ± 29 mg EAG/L, respectively), while the lowest concentration was observed in the Rosa María cultivar from Hidalgo (138 ± 86). These juices showed statistically significant differences with respect to all other cultivars of cactus pears studied (*p* < 0.05). Mean values for the purple, yellow and white cultivars of cactus pears were very similar; this indicates that TPC concentration does not correlate with the color of a group (Figure 4).

Stintzing *et. al.* [23] reported that the highest values of total phenolic compounds for red and purple cactus pears were 335 ± 19 and 600 ± 35 mg EAG/L, respectively; white cultivars of cactus pears showed a mean of 242 ± 13 mg EAG/L, and yellow cultivars of cactus pears showed an average of 247 ± 23 mg EAG/L. The yellow, white and a few of the red cultivars of Mexican cactus pear analyzed in this study showed slightly higher mean values than those previously reported. Furthermore, the highest concentration of TPC was found in the two cultivars of read cactus pears instead of the purple cultivars, as reported in other studies.

There was no significant difference (*p* < 0.05) between some of the juices of the same cactus pear cultivars and from different states (e.g., red cactus pear Vigor from Puebla and Hidalgo). However, there was significant difference in TPC between red cactus pear Lisa from Guanajuato and Zacatecas (*p* < 0.05). These differences could be related to the effect of the genotype, both of species and cultivars, as well as the crop conditions [33,34].

### 3.4. Analysis of Antiradical Activity Using the DPPH• Method

**Figure 5:**
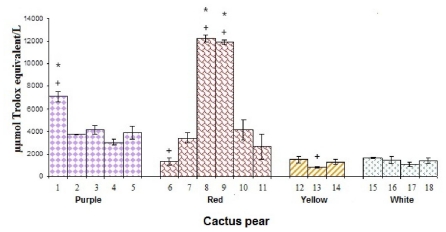
Antirradical activity of cactus pear studied cultivars. * denotes statistical significance at *p* < 0.05 among all studied cultivars of cactus pears. + indicates statistical significance at *p* < 0.05 among the cultivars of cactus pears in the same color.

The means of the antiradical activities between yellow and white cactus pear cultivars were not significantly different (*p* < 0.05) and were lower than the average antiradical activities from red and purple cultivars. The fruits with the highest antiradical activity were red cactus pear Lirio and Lisa from Zacatecas, with a mean activity over nine times higher than white, yellow and red cultivars from Hidalgo (Figure 5).

Among purple cactus pears, the highest antiradical activity was observed in fruit from Hidalgo, with 7103 ± 467 μmol ET/L, significantly higher than any other studied purple cultivar (*p* < 0.05). Antiradical activity measurements with DPPH• for red cactus pears was significantly correlated (*p* < 0.05) to the concentration of total phenolic compounds (*R*^2^ = 0.90) and ascorbic acid (*R*^2^ = 0.86). There was no significant correlation (*p* < 0.05) between the antiradical activity and TPC and CAA for the other studied cultivars. In the whole case there was no significant correlation between the free radical scavenging activity and betalain content.

Kuti [12] reported that red and purple cultivars showed higher antioxidant activities with respect to the white and yellow cultivars. As in other fruits, the total antiradical activity in cactus pears results from the sum of the antiradical activities of the substances that make up its composition, such as phenolic compounds, taurine, vitamins, betalains, and ascorbic acid.

### 3.5. Analysis of Antioxidant Activity by the β-Carotene and Linoleic Acid Method

In this analysis, the antioxidant activity in the juices of cactus pears was evaluated according to its protective effect on the discoloration of a β-carotene-linoleic acid emulsion. Therefore, this information is complementary to the above reported free radical scavenging activity.

**Figure 6:**
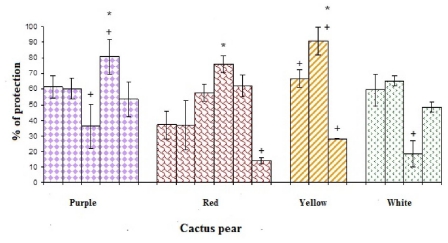
Antioxidant activity by the β-carotene and linoleic acid method of cactus pear studied cultivars. * denotes statistical significance at *p* < 0.05 among all studied cultivars of cactus pears. + indicates statistical significance at *p* < 0.05 among the cultivars of cactus pears in the same color.

Significant differences (*p* < 0.05) between the antioxidant activities within each color group of cactus pears studied were observed (Figure 6), yellow cactus pear Huesona from Zacatecas, red cactus pear San Martín from Hidalgo, and red cactus pear Lisa from Zacatecas showed the highest percentages of protection with 91 ± 9, 81 ± 11 and 76 ± 5%, respectively, while the lowest percentages were observed in red cactus pear Rosa María with 14 ± 2%, and white cactus pear Reina with 18 ± 8%, both from Hidalgo.

There were no significant differences (*p* < 0.05) between the purple (Púrpura from Hidalgo, Torreoja from Jalisco), red (San Martín from Puebla, Lirio from Zacatecas, and Lisa from Guanajuato), yellow (Picochulo from Zacatecas) and white (Reina from the State of Mexico and from Puebla) cactus pear cultivars, all this cactus pear colors yielded similar values in the protection of β-carotene emulsion. There were no significant correlations (*p* < 0.05) with other determinations made in this work, possibly due to the nature of reaction media; in this case, an oily medium analogous to the soluble nature of lipid membranes was used, while the ability to scavenge free radicals using DPPH• involves an aqueous medium. The percentage of protection to β-carotene emulsion in all samples was likely related to a synergistic effect of the compounds present in the composition of the fruit in an oily medium.

### 3.6. Chelating Activity of Ferrous Ions

It is well known that iron plays a central role in generating harmful oxygen species. Its redox cycling, in fact, promotes the Fenton reaction in which in the presence of O_2_ and transition metal ions, H_2_O_2_ can generate the very reactive hydroxyl radical [35]. Chelating activity results from the sum of the compounds present in the cactus pear that have been reported to have this activity. The phenolic compounds can react as metal chelating agents; they can extract iron ions and hinder radical reactions otherwise set into motion by the metal redox cycling [36–38].

**Figure 7:**
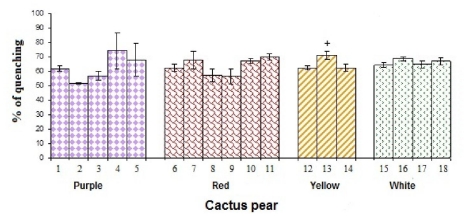
Chelating activity of cactus pear studied cultivars. * denotes statistical significance at *p* < 0.05 among all studied cultivars of cactus pears. + indicates statistical significance at *p* < 0.05 among the cultivars of cactus pears in the same color.

Figure 7 shows the chelating activity of the studied cultivars of cactus pears. It should be noted that the purple cactus pear Torreoja from Jalisco (51 ± 0.6%) shows the lowest percentage of chelating activity, whereas the purple cultivar San Martín from Hidalgo has the highest percentage of chelating activity (74 ± 12%). This activity did not significantly differ (*p* < 0.05) to the other studied cactus pear cultivars; therefore, it was concluded that chelating activity does not depend on the color of the cactus pear. No other significant correlations with any determination in this work were observed. It is noteworthy that no other study has reported chelating activity in cactus pears.

### 3.7. Stability of Betacyanin Pigments in the Presence of Hydroxyl Radicals (OH•)

**Figure 8:**
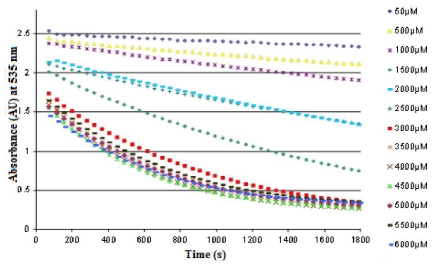
Kinetic curves for the speed of bleaching of betacyanin in the purple cactus pear Profundo from Hidalgo, based on the concentration of CuSO_4_.

Figure 8 shows an example of the effect of CuSO_4_ concentration on the speed of bleaching of the purple cactus pear Profundo from Hidalgo. The speed of bleaching of betacyanin is dependent on the concentration of Cu^2+^ and it increases as the concentration of the OH• radical increases. A kinetic curve for the bleaching of betacyanin in the presence of OH• radical was obtained. Bleaching kinetics were adjusted to the exponential function, and function *y* = Ae^−b^*^x^* was fitted to the curve to calculate the speed of bleaching of betacyanin (b), based on the concentration of CuSO_4_.

The concentration of CuSO_4_ that can be added to the juice without significantly increasing the speed of bleaching of betacyanin was related to the concentration of betacyanins and other compounds with antioxidant activities present in the juice (such as ascorbic acid and total phenolic compounds) since these antioxidant systems are able to protect betacyanins from oxidation.

**Figure 9:**
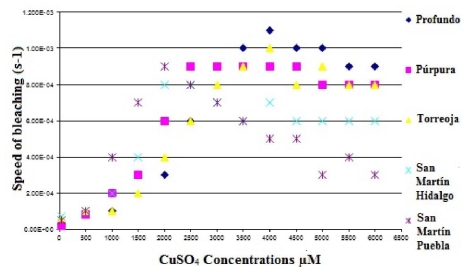
Speed of bleaching of betacyanin in the purple cactus pear cultivars as a function of the CuSO_4_ concentration.

**Figure 10:**
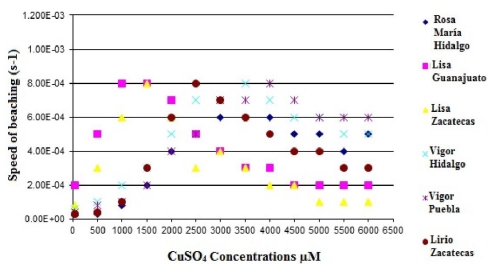
Speed of bleaching of betacyanin in the read cactus pear cultivars as a function of the CuSO_4_ concentration.

Figures 9 and 10 show the speed of bleaching of betacyanin in the purple and red cactus pear as a function of the CuSO_4_ concentration. It is important to mention that the extreme oxidizing conditions used highlight the great stability of the purple and red cactus pears when exposed to OH−.

For the purple cactus pear cultivars, a significant correlation (*p* < 0.01) exists between the maximum concentrations of CuSO_4_ that can be added to the juice without significantly increasing the speed of bleaching of betacyanin and the concentration of betacyanins (*R*^2^ = 0.58) and ascorbic acid (*R*^2^ = 0.92). On the other hand, for the red cactus pear cultivars, a significant correlation (*p* < 0.01) exists between this maximum concentrations of CuSO_4_ applied and betacyanin concentrations (*R*^2^ = 0.84).

**Figure 11:**
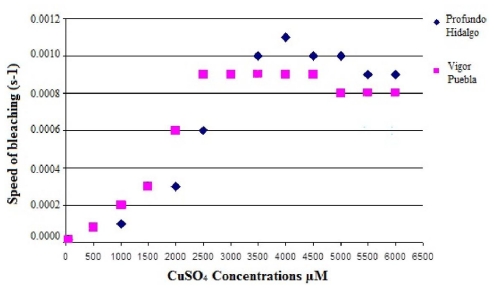
The bleaching speed (s-1) of purple cactus pear Profundo from Hidalgo and red cactus pear Vigor from Puebla, based on the concentration of CuSO_4_.

In certain purple cactus pear cultivars, it was necessary to use a higher concentration of CuSO_4_ compared to the red cactus pear cultivars (up to 4000 μM) to significantly increase the speed of bleaching. This is shown in Figure 11, where the speed of bleaching of purple cactus pear Profundo from Hidalgo and red cactus pear Vigor from Puebla were displayed as a function of the CuSO_4_ concentration. It is noteworthy that in both cultivars the antiradical activity measured by the DPPH• method did not present significant differences. Nevertheless, betacyanins concentration was greater in purple cactus pear Profundo from Hidalgo than in red cactus pear Vigor from Puebla.

## 4. Conclusions

It was observed that between same color cactus pear cultivars, but from a different state, the values obtained in some determinations were significantly different. Such differences could be linked to crop conditions, as cacti growing under limited conditions of soil and water could change their composition. Mean pH and °Bx values obtained indicate a good degree of maturity and commercial quality in the cultivars of cactus pears studied. Mean antiradical activities between yellow and white cactus pear cultivars were not significantly different and were lower than the average antiradical activities from red and purple cultivars. The free radical scavenging activity of the red cactus pears was significantly correlated to the concentration of total phenolic compounds and ascorbic acid. All 18 cultivars of cactus pears studied showed significant chelating activity of ferrous ions and the red and purple cactus pears showed a great stability when exposed to OH•.

The results of this research support the extensive use of the cactus pear cultivars showing higher antioxidant activity as high value-added ingredients in the food industry. Cactus pear juice may act as a powerful natural antioxidant, and could be an important additive for functional foods.

## Figures and Tables

**Table 1:** Description of 18 cultivars of cactus pear from six Mexican states.

No.	Cultivar	Mexican state	°Bx *	pH *
	**Purple cactus pears**			
1	Púrpura	Hidalgo	13.1 ± 1.1 ^a,b^	6.17 ± 0.031 ^b,c,d,e,f^
2	Torreoja	Jalisco	13.8 ± 0.3 ^a,b,c^	6.05 ± 0.015 ^b^
3	Profundo	Hidalgo	13.6 ± 0.6 ^a,b,c^	6.19 ± 0.080 ^b,c,d,e,f^
4	San Martín	Hidalgo	13.4 ± 0.2 ^a,b,c^	5.82 ± 0.119 ^a^
5	San Martín	Puebla	13.0 ± 0.8 ^a,b^	6.22 ± 0.079 ^c,d,e,f^
	**Red cactus pears**			
6	Vigor	Hidalgo	13.3 ± 0.7 ^a,b^	6.11 ± 0.051 ^b,c,d^
7	Vigor	Puebla	13.4 ± 0.5 ^a,b,c^	6.08 ± 0.017 ^b,c^
8	Lirio	Zacatecas	14.0 ± 0.3 ^a,b,c^	6.16 ± 0.012 ^b,c,d,e^
9	Lisa	Zacatecas	15.1 ± 0.1 ^d^	6.20 ± 0.072 ^b,c,d,e,f^
10	Lisa	Guanajuato	14.0 ± 0.1 ^a,b,c^	6.29 ± 0.085 ^e,f^
11	Rosa María	Hidalgo	13.6 ± 0.2 ^a,b,c^	6.26 ± 0.017 ^d,e,f^
	**Yellow cactus pears**			
12	Picochulo	Zacatecas	14.8 ± 0.5 ^c,d^	6.11 ± 0.081 ^b,c^
13	Huesona	Zacatecas	13.6 ± 0.3 ^a,b,c^	6.22 ± 0.055 ^c,d,e,f^
14	Milpa Alta	Hidalgo	13.7 ± 1.4 ^a,b,c^	6.17 ± 0.061 ^b,c,d,e,f^
	**White cactus pears**			
15	Reina	Estado de México	14.4 ± 0.5 ^b,c,d^	6.16 ± 0.070 ^b,c,d,e^
16	Reina	Puebla	13.1 ± 0.3 ^a,b^	6.20 ± 0.090 ^b,c,d,e,f^
17	Reina	Hidalgo	12.8 ± 1.6 ^a^	6.28 ± 0.072 ^e,f^
18	Cristalina	Puebla	13.5 ± 0.4 ^a,b,c^	6.32 ± 0.176 ^f^

* Data with the same lowercase letter are not significantly different (*p* < 0.05).
